# Muscle cells challenged with saturated fatty acids mount an autonomous inflammatory response that activates macrophages

**DOI:** 10.1186/1478-811X-10-30

**Published:** 2012-10-19

**Authors:** Nicolas J Pillon, Karen Arane, Philip J Bilan, Tim T Chiu, Amira Klip

**Affiliations:** 1 Program in Cell Biology, The Hospital for Sick Children,Toronto, Ontario, M5G 1X8, Canada

**Keywords:** Inflammation, Muscle, Palmitate, Palmitoleate, Saturated fat, Obesity, Insulin resistance, GLUT4, Glucose uptake

## Abstract

Obesity is associated with chronic low-grade inflammation. Within adipose tissue of mice fed a high fat diet, resident and infiltrating macrophages assume a pro-inflammatory phenotype characterized by the production of cytokines which in turn impact on the surrounding tissue. However, inflammation is not restricted to adipose tissue and high fat-feeding is responsible for a significant increase in pro-inflammatory cytokine expression in muscle. Although skeletal muscle is the major disposer of dietary glucose and a major determinant of glycemia, the origin and consequence of muscle inflammation in the development of insulin resistance are poorly understood.

We used a cell culture approach to investigate the vectorial crosstalk between muscle cells and macrophages upon exposure to physiological, low levels of saturated and unsaturated fatty acids. Inflammatory pathway activation and cytokine expression were analyzed in L6 muscle cells expressing myc-tagged GLUT4 (L6GLUT4*myc*) exposed to 0.2 mM palmitate or palmitoleate. Conditioned media thereof, free of fatty acids, were then tested for their ability to activate RAW264.7 macrophages.

Palmitate -but not palmitoleate- induced IL-6, TNFα and CCL2 expression in muscle cells, through activation of the NF-κB pathway. Palmitate (0.2 mM) alone did not induce insulin resistance in muscle cells, yet conditioned media from palmitate-challenged muscle cells selectively activated macrophages towards a pro-inflammatory phenotype.

These results demonstrate that low concentrations of palmitate activate autonomous inflammation in muscle cells to release factors that turn macrophages pro-inflammatory. We hypothesize that saturated fat-induced, low-grade muscle cell inflammation may trigger resident skeletal muscle macrophage polarization, possibly contributing to insulin resistance *in vivo*.

## Lay abstract

Obesity is associated with chronic activation of the immune system. In response to high fat diet, the fat tissue attracts immune cells that cause low, sustained inflammation responsible for making the body resistant to insulin. Recent studies show that inflammation also happens in muscle, but its origin and consequence for the development of type 2 diabetes are not understood.

We used cells in culture to investigate the communication between muscle and immune cells upon exposure to low levels of a saturated fatty acid (palmitate as in western diet) or an unsaturated fatty acid (palmitoleate, as in Mediterranean diet). We analyzed the effects of these fatty acids on muscle inflammation and next collected the solution surrounding these cells (called conditioned media), and tested its ability to activate immune cells.

Palmitate -but not palmitoleate- induced inflammation in muscle cells but the low dose used (0.2 mM) alone did not make muscle cells resistant to insulin. Strikingly, conditioned media from palmitate-challenged muscle cells selectively made macrophages acquire a pro-inflammatory phenotype.

These results provide direct evidence of a muscle-to-immune cell communication in the context of fat exposure and suggest that this communication might occur in the body. This is of importance as fighting muscle inflammation could be a therapeutic strategy to prevent type 2 diabetes.

## Background

One and a half billion people world-wide are overweight [1], and this condition is a leading cause of type 2 diabetes [2]. High caloric diet and in particular consumption of saturated fatty acids increase the likelihood of developing obesity. These conditions beget whole-body insulin resistance, a cornerstone of the metabolic syndrome, and obesity-induced insulin resistance is a major risk factor in the development of type 2 diabetes [3]. Palmitic acid (hexadecanoic acid, 16:0) is the most common saturated fatty acid in the western diet, and a major constituent of the total non-esterified fatty acids in the blood [4,5]. In cell and animal studies, palmitate leads to the development of insulin resistance and inflammation [6-8], whereas unsaturated fatty acids are often beneficial or at least less deleterious. Indeed, the monounsaturated fatty acid palmitoleic acid ((*Z*)-9-hexadecenoic acid, 16:1Δ9), which differs from palmitate by the presence of one double-bond, increases insulin sensitivity and suppresses inflammation [9,10].

Adipose tissue expansion in response to high fat diet (HFD) is accompanied by a local, low-grade inflammation [11-13]. *In vivo*, inflammation may be triggered by adipocytes and/or endothelial cells, putatively through the release of cytokines and other paracrine factors [14,15]. However, it is the resident macrophages and infiltrating macrophage-like cells that assume a pro-inflammatory phenotype, contributing to the brunt of pro-inflammatory cytokine production (e.g., TNFα, IL-6 and IL-1β) within adipose tissue [16,17]. Whereas the initial trigger of inflammation in the adipose tissue is debated, high levels of palmitate and other saturated fats promote a pro-inflammatory phenotype in macrophages *in vitro*[18,19]. In turn, inflamed immune cell populations can adversely affect the metabolic function of adipose tissue; and indeed, inflammation *per se* can impair insulin action in adipocytes, reducing lipogenesis and enhancing lipolysis [15,20].

In spite of the pivotal role of macrophages in the development of insulin resistance within adipose tissue during HFD, inflammation is not restricted to this tissue. Indeed, HFD is responsible for a significant increase in the expression of the pro-inflammatory cytokines TNFα, IL-6 and IL-1β in skeletal muscle [21]. This is important because skeletal muscle is the major disposer of dietary insulin and a major determinant of glycemia, and whole-body insulin resistance arises only when skeletal muscle and/or the liver become resistant to the actions of insulin [22]. In addition, resident macrophages are a constitutive component of skeletal muscle, relevant for inflammatory responses associated with muscle injury, dystrophies and endotoxemia [23,24]. Notably, we and others recently detected increased gene expression of F4/80 (macrophage marker) and CD11c^+^ (pro-inflammatory macrophage- or dendritic cell-like) in muscle from high fat-fed mice [12,25]. Moreover, upon extraction and flow cytometry, we detected a population of F4/80/CD11^+^ cells (inflammatory macrophages), and others have observed macrophages in fat depots within muscle that expand under HFD and obesity [13,26]. Notably, macrophages increase in number within skeletal muscle from obese subjects and their number and inflammatory phenotype correlate positively with body mass index and negatively with insulin sensitivity [25,27]. Compellingly, media collected from saturated fatty acid-treated macrophages confer insulin resistance to muscle cells in culture [12,14,18].

While those studies provide pieces of evidence of communication from macrophages to muscle cells, they did not examine the communication from muscle cells to macrophages, and the reason for macrophages turning inflammatory in the muscle (or adipose) tissue milieu is unknown. Conceivably, muscle cells on their own generate cues that can impact on macrophages. The major aim of this study was to test the hypothesis that low levels of saturated fatty acids evoke inflammatory responses in muscle cells that may in turn affect the macrophage phenotype. A cell culture model was used to provide proof-of-principle of muscle to macrophage communication, as it avoids the complexities expected from a whole-body analysis. The results demonstrate that, at low doses, palmitate activates inflammatory pathways within muscle cells leading to the expression of inflammatory cytokines, and media collected from these cells shifts macrophages towards a pro-inflammatory mode. Conversely, the unsaturated palmitoleate did not activate inflammatory pathways in muscle cells, and media thereof did not confer a pro-inflammatory phenotype to macrophages. These results provide direct evidence of a muscle to macrophage communication in the context of exposure to saturated fat.

## Results

Skeletal muscle is composed primarily of muscle fibers, but also encompasses blood and lymph vessels, nerves and immune cells. As each of these cell types can potentially respond to a high fat environment *in vivo*, we chose a cell culture approach to investigate the specific crosstalk between muscle cells and macrophages in the context of fatty acid exposure. This strategy enables us to control individual variables, to determine vectorial communication, and to explore separately the responses of each cell type. L6GLUT4*myc* cells were used as a prototypic muscle cell system that responds to insulin [28] while RAW264.7 macrophages were used as they constitute a well-characterized macrophage line [12]. Palmitate was used as a typical saturated fatty acid found in western diets and palmitoleate was used to decipher the contribution of acyl chain saturation in palmitate. From healthy to obese individuals, total free fatty acid concentration in serum can range from 0.2 to 0.8 mmol/L, composed of roughly 25% palmitate and 4% palmitoleate [5,29,30]. Consequently, palmitate can reach up to 0.2 mM in obese individuals [4], which is the concentration we chose to use in our study. Each fatty acid was conjugated to low endotoxin and lipid-free BSA, and control treatment with BSA was carried out in parallel. BSA and fatty acid-BSA were removed from the muscle cultures prior to further incubation to generate the respective conditioned media, and non-esterified free fatty acids were undetectable in such conditioned media (Table 1). These fatty acid-free conditioned media are hereafter called CM-PA (collected from muscle cells pretreated with PA); CM-PO (from muscle cells pretreated with PO), or CM-BSA (from muscle cells pretreated only with BSA).

**Table 1:** NEFA concentration

	**Initial**	**CM**
RM	*25* ± 25	*< 10*
BSA	15 ± 14	*< 10*
PA	197 ± 58	*< 10*
PO	252 ± 80	*< 10*

NEFA concentration in the initial solution and conditioned media (CM) was measured using the enzymatic method based on Acyl-CoA oxidase as described in methods. The measurable range of the kit is 0.01-4.0 mM. Results are means ± SD expressed in micromoles/liter from at least 4 independent experiments (n≥4).

### Low-dose palmitate up-regulates cytokine gene expression in muscle cells

**Figure 1:**
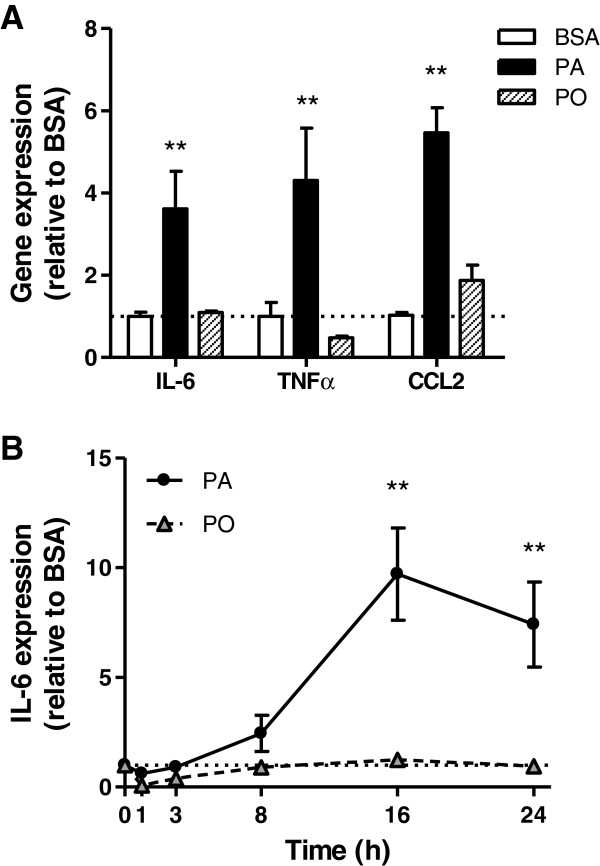
**Low level palmitate treatment activates inflammatory cytokine & chemokine gene expression in muscle cells.** L6 muscle cells were treated with 0.2 mM palmitate (PA), palmitoleate (PO) or BSA control for (A) 24 h or (B) at the times indicated. **A**) mRNA expression was analyzed by qRT-PCR using primers for TNFα IL-6 and CCL2. **B**) mRNA expression was analyzed by qRT-PCR using primers for IL-6. Results are normalized to the reference gene hprt1 and then to the BSA control and reported as fold change ±SEM (n≥3), **p<0.01 vs BSA control (1-way ANOVA).

Gene expression by muscle cells of a number of cytokines was measured by qPCR. IL-6 was the most abundantly expressed cytokine in muscle cells, relative to a housekeeping control gene, whereas TNFα expression was barely detectable and IL-1β was undetectable (data not shown). Treatment with palmitate -but not palmitoleate- increased IL-6 and TNFα gene expression by 3.8- and 4-fold, respectively, compared to the BSA control (Figure 1A) These results suggest that TNFα and IL-6 are *bona fide* muscle cell products up-regulated in response to a palmitate challenge. Accordingly, we used IL-6 as the prototypical cytokine produced by muscle under a palmitate challenge. IL-6 expression was induced by palmitate in a time-dependent manner measured for up to 24 h (Figure 1B).

In an attempt to characterize the molecules released by muscle cells present in the conditioned media (CM), we used a protein array that was able to detect several cytokines and chemokines (Additional file 1: Figure S1). Attention was paid to expose the CM-BSA and CM-PA arrays for precisely the same time. Notably, PDGF levels were decreased in the CM-PA array, while there was an overall elevation in several other cytokines, demonstrating that differences observed between the two samples are not generic. The most abundant cytokines and factors within either CM revealed by this approach were CINC1 (CXCL1), PDGF, VEGF and TIMP1. TIMP1 is an inhibitor of matrix metalloproteinases, which is also involved cell proliferation and apoptosis. VEGF and PDGF are growth factors involved in vasculogenesis and angiogenesis and CINC1 (CXCL1) promotes neutrophil migration. None of these factors have been shown to induce inflammation in macrophages.

Although small, there were discernible elevations in IL-6 and CCL2 levels in the CM-PA compared to CM-PO array, consistent with the detectable rises in their mRNA levels in the corresponding cells (Figure 1 and Additional file 1: Figure S1). On the other hand, there was no detectable TNFα protein (Additional file 1: Figure S1), and this is also consistent with the very low mRNA expression detected in the cell lysates. Other proteins that were clearly higher in CM-PA compared to CM-PO included activin A, agrin, INFγ, IL-1α and MIP3α.

### Signalling pathways activated by low-dose palmitate

**Figure 2:**
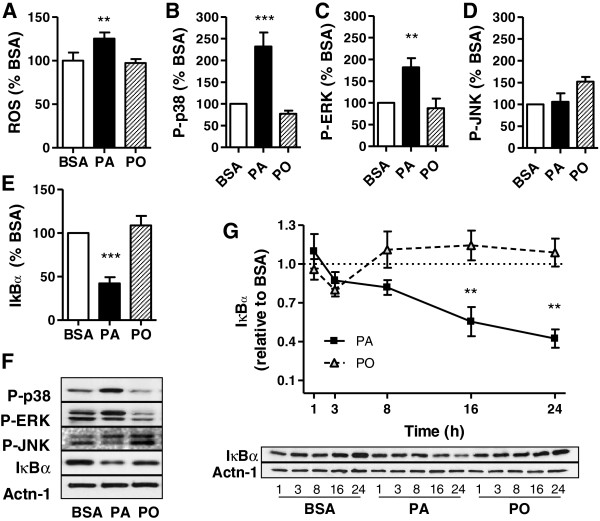
**Palmitate activates ROS and signalling pathways in muscle cells.** L6 muscle cells were treated for 24h with 0.2 mM palmitate (PA), palmitoleate (PO) or BSA control. **A**) Reactive oxygen species were analyzed using DCFDA, normalized to the protein content and expressed as percent of the BSA control. Results are mean ±SEM (n=4) analyzed using 1-way ANOVA, **p<0.01 vs BSA control. **B**-**E**) Proteins were extracted with standard lysis buffer and analyzed by western blotting using specific antibodies to IκBα and the phosphorylated forms of ERK, JNK and p38 MAPK. Results were normalized to the loading control actinin-1, expressed relative to the BSA control as mean ±SEM (n=4) and analyzed using 1-way ANOVA, **p<0.01, ***p<0.001 vs BSA control. **F**) Representative blots for B-E. **G**) Time-course of IκBα degradation. The changes of IκBα were calculated to the BSA control as indicated by the dotted line set at 1.0 from the y-axis **p<0.01 vs BSA control.

Obesity and type 2 diabetes are associated with activation of stress kinases, as well as activation of the canonical NF-κB inflammation pathway in several tissues [31]. IκBα degradation is necessary for NF-κB migration to the nucleus to initiate cytokine transcription. Mitogen Activated Protein Kinases (MAPK) and Reactive Oxygen Species (ROS) are known activators of this pathway [32]. In L6 muscle cells, palmitate treatment significantly increased ROS levels by 25% (Figure 2A), and caused phosphorylation of Extracellular Regulated Kinase 1/2 (ERK1/2) and p38 MAPK, whereas JNK was not phosphorylated (Figure 2B-D and Figure 2F). In contrast to palmitate, palmitoleate did not significantly activate any of these stress kinases or ROS production, although JNK phosphorylation tended to be higher. Palmitate also caused significant activation of the NF-κB pathway, revealed by a 50% reduction in IκBα mass (Figure 2E-F) that was time-dependent, reaching its lowest point by 24 h of treatment (Figure 2G).

**Figure 3:**
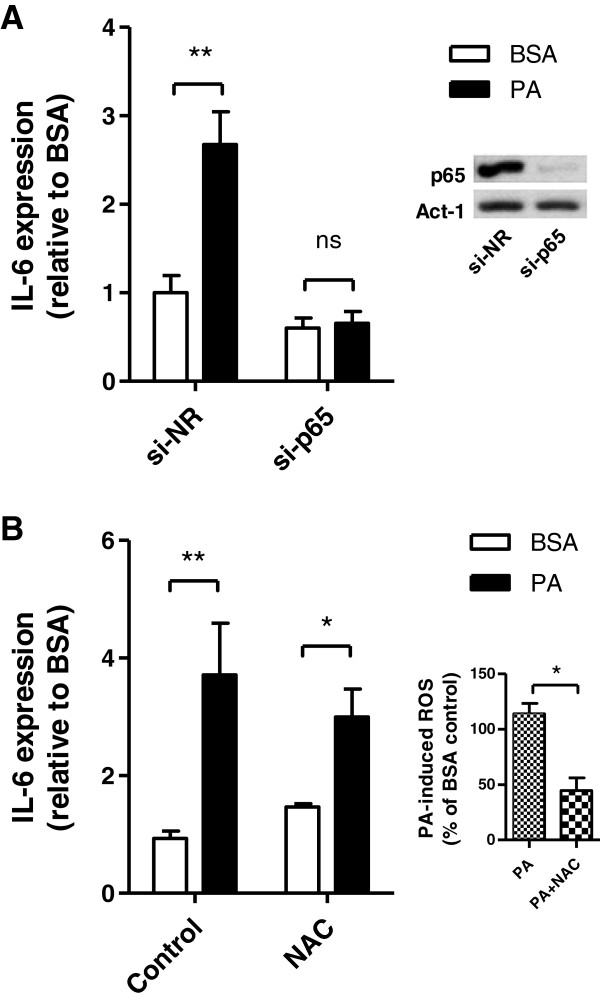
**Palmitate-induced cytokine expression occurs through NF-κB pathway. A**) NF-κB was silenced using specific siRNA to the p65 subunit (p65) or control non-related siRNA (NR) before treatment of cells for 24h with 0.2 mM palmitate or BSA. **B**) Cells were incubated in the presence of 0.2 mM palmitate or BSA without or with N-acetyl-cysteine (5 mM) for 24 h. Inset: ROS production was measured as described in Figure 2A and reported as percent of the BSA control. All results were normalized to BSA control and reported as fold change ± SD from at least 3 independent experiments (n≥3). *p<0.05, **p<0.01, ns=non significant.

To explore the involvement of the NF-κB pathway in palmitate-induced cytokine expression, we silenced the expression of the p65 subunit of the NF-κB complex via siRNA oligonucleotides. In cells depleted of p65 protein by 70%, palmitate-induced expression of IL-6 was abolished, whereas a control non-related siRNA failed to diminish the palmitate effect (Figure 3A). Silencing p65 also prevented the palmitate-induced rise in TNFα and CCL2 expression (Additional file 1: Figure S2). On the other hand, although palmitate stimulated ROS production in muscle cells, the rise in cytokine expression caused by palmitate was not prevented by the antioxidant N-acetyl-cysteine despite its clear ability to lower ROS levels (Figure 3B). Hence, ROS are unlikely to be a signal for the cytokine expression by muscle cells evoked by the fatty acid.

### Low-dose palmitate does not confer insulin resistance to muscle cells

**Figure 4:**
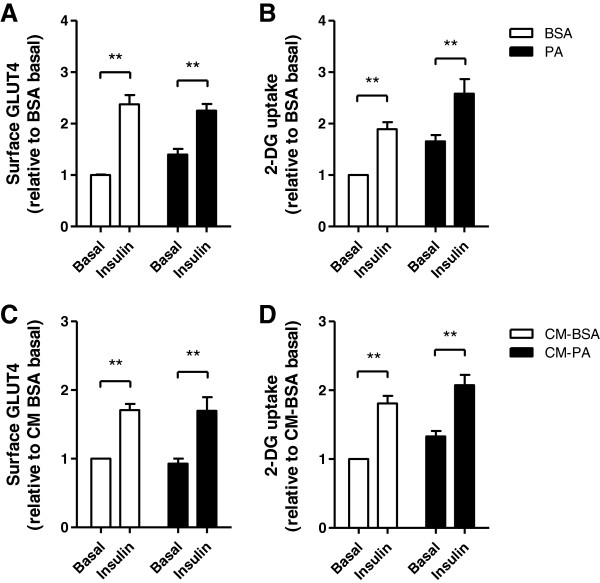
**Palmitate treatment does not induce insulin-resistance in muscle cells. A-B**) The direct effect of PA was measured by treating L6 cells with 0.2 mM palmitate (PA), palmitoleate (PO) or BSA control for 24 h, followed by 3 h serum starvation and insulin stimulation (20 min, 100 nM) in the absence of PA. **C**-**D**) The indirect effect of palmitate through a possible autocrine effect was tested by generating conditioned media from muscle and applying it on naive untreated muscle cells. Surface GLUT4*myc* and glucose uptake were measured as described in Methods. Results are normalized to the CM-BSA control and expressed as mean ± SEM (n=4), **p<0.01.

The above results show that physiological levels of palmitate (0.2 mM) activated stress and inflammatory pathways in muscle cells. Because much elevated levels of palmitate (0.5 mM and higher) can induce insulin resistance in muscle cells, in part through the generation of oxidative stress [6,33,34], we explored if this would similarly occur in response to low palmitate. Interestingly, though 24 h incubation of muscle cells with 0.2 mM palmitate activated NF-κB signalling and IL-6 expression (shown above, Figures 1, 2, and 3), this did not diminish the classical insulin responses of GLUT4 translocation (Figure 4A) and in fact, increased glucose uptake (Figure 4B). At this low palmitate exposure, insulin stimulated GLUT4 translocation by 2.3-fold and glucose uptake by 60%, comparable to the response of BSA-treated control cells. Hence, the milder lipid exposure can activate inflammation, but is not sufficient on its own to cause insulin resistance in isolated muscle cells.

As conditioned media from palmitate-treated muscle cells (CM-PA) contain several cytokines and chemokines that might provoke insulin-resistance in muscle cells as shown in other studies [35-37], we also assessed whether such CM would exert an autocrine insulin resistance effect, independently of any direct metabolic effect of palmitate. To this end, CM-PA or CM-BSA were applied to sets of naive L6 cells for 24 h. Following incubation, insulin stimulated GLUT4 translocation by 70% in both conditions (Figure 4C) and glucose uptake by 80% and 60%, respectively (Figure 4D). There was no statistical difference between the responses to CM-BSA and CM-PA, indicating the absence of muscle-to-muscle autocrine transmission of insulin-resistance.

### Conditioned medium from muscle cells promotes macrophage inflammation

**Figure 5:**
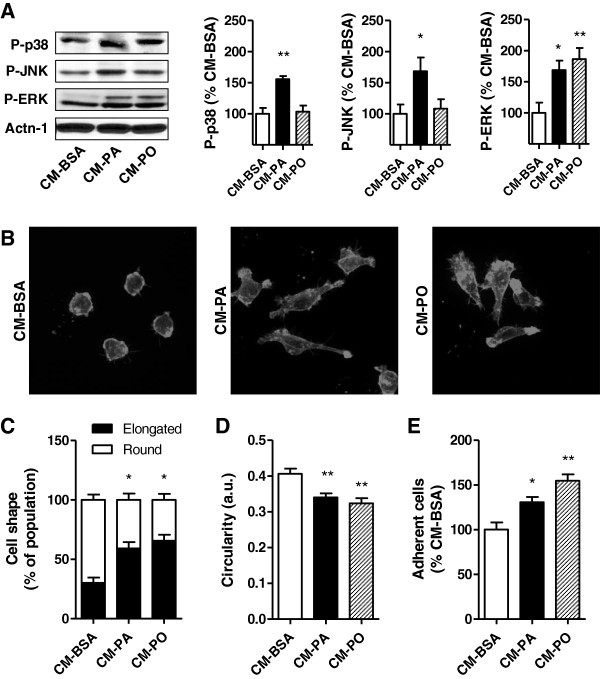
**Muscle cell conditioned media induce macrophage spreading and adhesion.** Conditioned media (CM) from myoblasts treated with 0.2 mM palmitate was applied on RAW264.7 macrophages for 2 h. **A**) Proteins were extracted and analyzed by western blotting using specific phospho antibodies to ERK, JNK and p38 as previously described. **B**-**C**) Actin labelling with rhodamine phalloidin and cell shape quantification were performed as described in Methods. **E**) Circularity in images was quantified with ImageJ software. **E**) Measurement of RAW264.7 cell adhesion in response to CM, as described in Methods. Results were normalized to BSA control, reported as fold change ± SD and analyzed using 1-way ANOVA, n≥3, *p< 0.05 , **p<0.01.

Given the increased gene expression of cytokines and chemokines in palmitate-treated muscle cells and the elevated levels in CM-PA, we explored whether this medium could affect macrophage function and phenotype. Activated macrophages are characterized by stimulation of the MAPK signalling pathways [38], as well as actin filament remodelling extending filopodia and lamellipodia that promote cell spreading [39]. RAW macrophages responded to CM-PA with a rise in phosphorylation of ERK, p38 MAPK and JNK (Figure 5A). In contrast, CM-PO only provoked ERK phosphorylation. Macrophages treated with either CM-PA or CM-PO assumed an elongated shape visualized upon actin filament decoration with rhodamine-phalloidin (Figure 5B-D), and showed heightened adherence to the substratum, compared to CM-BSA-treated controls (Figure 5E).

**Figure 6:**
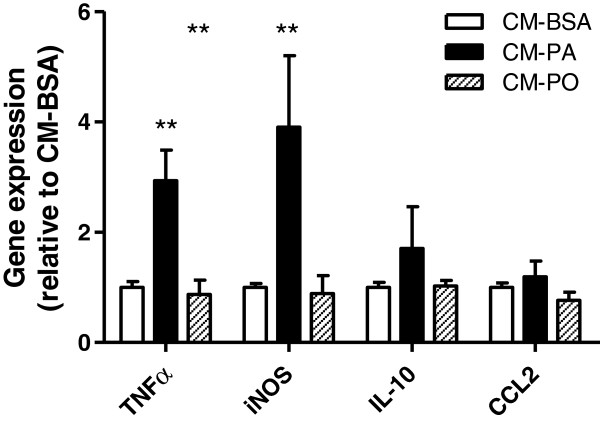
**Muscle cell conditioned media induce macrophage inflammation.** Conditioned media (CM) from myoblasts treated with 0.2 mM palmitate was applied on RAW264.7 macrophages for 6 h and gene expression in RNA of the latter was measured by qPCR as described in Methods. Results were normalized to the BSA control, reported as fold change ± SD and analyzed using 1-way ANOVA, n≥3, **p< 0.01.

Inflammatory macrophages are typified by elevated gene expression of the pro-inflammatory cytokine TNFα and the enzyme iNOS, with converse diminished gene expression of the anti-inflammatory cytokine IL-10 [40]. Macrophages exposed to CM-PA exhibited an increased expression of TNFα and iNOS mRNA but no significant change in IL-10 and CCL2 mRNA (Figure 6). On the other hand, CM-PO did not affect the expression of any of these genes.

Since the analysis of the CM (Additional file 1: Figure S1) revealed that many changes were modest, this suggested that the active component is either not detected by this array, that molecules other than cyto/chemokines contribute, and/or that a complex mechanism involving synergistic effects of low levels of several cytokines and chemokines may participate in conferring macrophage polarization. Therefore, as preamble, we ascertained whether proteins mediate this cell-cell communication. CM were boiled (95°C, 15 minutes) or treated with proteinase K (100 μg/mL, 2 h) to digest proteins, and the enzyme itself was then heat inactivated. Both of these strategies successfully blunted induction of TNFα mRNA expression within macrophages provoked by muscle CM-PA (Additional file 1: Figure S3). This demonstrates that the factor(s) responsible for macrophage polarization of macrophages is (are) of protein nature. These results highlight a protein-mediated, muscle-to-macrophage communication that confers an inflammatory phenotype to the latter, when the former are pre-exposed to a saturated fatty acid.

## Discussion

Recent studies have detected elevated numbers of myeloid cell or macrophage markers in skeletal muscle during HFD and obesity [13,25-27], but how these immune cells are influenced by the surrounding tissue and their role in the progression of insulin resistance are unknown. Here we present evidence that muscle cells exposed to a physiological dose of the saturated fatty acid palmitate, activate their endogenous inflammatory programs and that conditioned-medium from such challenged muscle cells induces a pro-inflammatory phenotype in macrophages. In contrast to palmitate, the monounsaturated fatty acid palmitoleate had no significant effect on muscle cell autonomous inflammation or on the crosstalk from muscle cells to macrophages. Overall, these results lend support to the hypothesis that *in vivo*, skeletal muscle may contribute to the low-grade inflammation observed during high fat diet through the production of cytokines affecting immune cell responses. The latter could be either resident or recruited macrophages, and their bi-directional crosstalk with muscle cells might contribute to muscle insulin resistance during dyslipidemia or HFD earlier than the development of direct, lipid-induced insulin resistance.

### Low-dose palmitate elicits muscle cell-autonomous inflammation

Myoblasts treated with palmitate exhibit activation of pathways leading to inflammation, specifically activating MAPK, elevating ROS and inducing IκBα degradation (Figure 2). A similar response is observed in response to higher palmitate concentrations [41,42]. Low levels of palmitate also led to increased gene expression of several pro-inflammatory cytokines (TNFα, IL-6) and chemokines (CCL2), as well as greater release of several cytokines from muscle such as INFγ, IL-1α, IL-6, CCL2, TIMP1 and CXCL1, demonstrating the development of muscle cell-autonomous inflammation. This process was evident by 8 h of incubation with palmitate, and peaked by 16 h, evincing the activation not only of post-translational signalling, but also of transcriptional and metabolic events (i.e., ROS production). Interestingly, palmitoleate did not activate any of these pathways in the muscle cells, and did not change cytokine expression. The palmitate-induced increase in cytokine gene expression is not as impressive as that typically elicited by LPS, rather it is slow, sustained, and the fold-change is comparable to that reported in muscle from obese vs. lean mice [21].

TLR4 and TLR2 have been invoked to participate in some response to saturated fatty acids in cell culture and to HFD *in vivo*[43-45]. Indeed, TLR4 deficient mice exhibit decreased NF-κB activation in adipose tissue in response to a diet rich in palmitate [46], and inhibition or deletion of TLR4 confers partial protection against palmitate-induced NF-κB activation in rodent skeletal muscle [47]. However, other pattern recognition receptors such as Nod1 and Nod2 also funnel their signals through NF-κB. Muscle cells express these receptors, and we have found that agonist-induced activation of Nod2 causes IκBα degradation and TNFα expression in these cells [48]. Future work should explore whether TLRs, Nod1 or Nod2 participate in the inflammatory response of muscle cells induced by palmitate. The current study also shows that 0.2 mM palmitate increased muscle cell ROS levels (Figure 2A), as it occurs in response to higher palmitate concentrations [49]. Dampening such ROS levels with N-acetyl-cysteine failed to attenuate the palmitate-induced cytokine production, but we cannot rule out that the remaining ROS levels might be permissive for cytokine production under these conditions.

### Muscle cell-autonomous inflammation via NF-κB does not suffice to cause insulin resistance

The NF-κB pathway, which controls the expression of inflammatory cytokines, is involved in the pathogenesis of whole-body insulin resistance [50,51]. NF-κB activity is 2.7-fold higher in muscles from obese type 2 diabetic subjects compared to lean individuals [52] and this difference is conserved in human primary myocytes in culture derived from obese type 2 diabetic patients [50]. However, the cause for the activation is likely complex and may result from the combined input of signals derived from other cells on the muscle, along with the elevated cytokines and fatty acids in the circulation. Further, in muscle cell cultures, very high concentrations of palmitate (0.5-0.8 mM) activate novel-type protein kinase C’s in addition to the NF-κB pathway [41,42] and elevate the expression of IL-6, compromising insulin signalling and further causing GLUT4 down-regulation [41,53]. Here we present evidence that preventing muscle cell-autonomous activation of the NF-κB pathway via knockdown of the p65 NF-κB subunit eliminates the palmitate-induced upregulation of IL-6, TNFα and CCL2 expression.

Importantly, in spite of causing muscle cell-autonomous inflammation involving NF-κB signalling and cytokine production, low levels of palmitate did not provoke insulin resistance of GLUT4 translocation or glucose uptake. Hence, engagement of the NF-κB pathway *per se* is insufficient to alter the major insulin action in skeletal muscle cells. It is possible that this pathway would be relevant if other stress signals were activated, and notably palmitate did not cause JNK phosphorylation in muscle cells. Activation of this kinase is a typical feature of insulin resistant muscle in the context of HFD *in vivo*, and a recent study shows that muscle-specific transgenic expression of constitutively-active JNK *per se* confers insulin resistance [54]. Further, our results indicate that any cytokines present in CM from palmitate-treated muscle cells do not suffice to confer insulin resistance to naive muscle cells (i.e., muscle-to-muscle communication) while the same CM was able to activate macrophages (discussed below).

### Muscle cells confer macrophage pro-inflammatory polarization

A core result of this study is that muscle cells pre-treated with low-dose palmitate produce CM that makes macrophages assume a pro-inflammatory phenotype. Specifically, CM-PA induced activation in macrophages of selective MAPK signalling pathways (p38 and JNK), along with promoting cell spreading and gene expression of the pro-inflammatory markers TNFα and iNOS. These results render proof-of-concept that muscle cells can release factors communicating with immune cells in the context of physiological levels of saturated fat. On the other hand, CM from palmitoleate-treated muscle cells did not bring about these responses, suggesting that unsaturated fatty acids do not elicit muscle-to-macrophage crosstalk. Boiling and proteinase K treatments of CM-PA blunted the power of this medium to confer inflammation to macrophages, suggesting that the factor(s) released by muscle is (are) cytokine(s) or other proteins. Cytokine array analysis did not reveal any salient increases in cytokines in CM-PA compared to CM-PO, however the array is limited and it is also possible that levels below the detection limit of the array, or proteins not analyzed, confer polarization. A synergistic effect of low-doses of several different factors is also possible, and indeed in 3T3-L1 adipocytes, LPS, tumor necrosis factor-alpha (TNFα), or interferon-gamma (IFNγ) had to be combined to increase iNOS activity [55]. Further in-depth analysis, beyond the scope of the present study, will be necessary to identify the proteins and/or synergistic effects involved in the muscle-macrophage crosstalk described.

Of note, CM-PO resembled CM-PA in its ability to activate ERK in macrophages, and this response is consistent with the morphological change of macrophages induced by both media, characterized by cell spreading. ERK is involved in lamellipodia protrusion [56], and consistent with these observations, inhibiting ERK prevented the heightened macrophage adhesion elicited by either CM-PA or CM-PO (Additional file 1: Figure S4). Interestingly, cell spreading is a feature of both pro- and anti-inflammatory macrophages [57]. We therefore surmise that macrophage adhesion and pro-inflammatory activation in response to muscle cell CM are not obligatorily linked, as the two responses can be dissociated depending on the pretreatment of the muscle cells. These results illustrate that different nutrients promote a distinct muscle-to-macrophage crosstalk. Immune cells can be activated in muscle for beneficial outcomes such as resolving injury [58], and we hypothesize that CM-PO might counteract inflammatory cues acting on macrophages; future studies should explore this possibility.

In conclusion, we report that physiological levels of palmitate cause muscle cell-autonomous inflammation that, while not sufficing to cause insulin resistance within the muscle cells, result in production of proteins conferring a pro-inflammatory phenotype to macrophages in culture. If one may extrapolate, it is conceivable that muscle-induced macrophage polarization is one of the most sensitive responses to a saturated fat environment *in vivo*. Given the ability of pro-inflammatory macrophages to confer insulin resistance to muscle, we hypothesize that a feed-forward cycle of muscle-macrophage cross talk can contribute to the development and perpetuation of full-blown insulin resistance.

## Methods

### Reagents

Sodium palmitate, palmitoleate, BSA (low endotoxin), endotoxin-free water and protease inhibitor cocktail were from Sigma-Aldrich. Antibodies to phospho-JNK (Thr183/Tyr185), phospho-ERK (Thr202/Tyr204), phospho-p38 MAPK (Thr180/Tyr182), and IκBα were from Cell Signaling Technology (Beverly, MA). Control siRNA (siNR) and siRNA against p65 were purchased from Qiagen (Chatsworth, CA) and MYD88 inhibitory and control peptides were from Invivogen (San Diego, CA). 2-Deoxy-[^3^H]glucose was from Perkin Elmer.

### Fatty acid preparations

Solutions of 200 mM sodium palmitate or palmitoleate were prepared in 50% ethanol by heating at 55°C and vortexing until dissolved. These solutions were diluted 25-times in a 10% fatty acid-free, low-endotoxin BSA solution to achieve a final molar ratio of 5:1. Conjugation was done at 40°C for 2 h. BSA-fatty acid conjugates were further diluted 40-fold in cell culture media to reach a final concentration of 0.2 mM palmitate or palmitoleate. Control BSA was prepared by adding the same amount of 50% ethanol into a 10% BSA solution. All preparations were aliquoted and frozen at -20°C.

### Cell culture and transfection

Rat L6 muscle cells with stable expression of *myc*-tagged glucose transporter 4 (L6GLUT4*myc*), and RAW264.7 macrophages were each grown in α-MEM supplemented with 10% fetal bovine serum (FBS), 100 units/mL penicillin, 100 μg/mL streptomycin and 250 ng/mL amphotericin B. siRNAs oligonucleotides were transfected into the cells with calcium phosphate-based CellPhect Transfection Kit (GE Healthcare Bio-Sciences, Piscataway, NJ). Muscle cells were treated with 200 nM siRNA-calcium phosphate precipitates for 12-16 h before removal and maintained for 72 h until experiments.

### RNA isolation & PCR

RNA was isolated using Trizol (Invitrogen) and cDNA was generated by reverse transcription using the SuperScript® VILO™ cDNA kit (Invitrogen) according to the manufacturer's instructions. Fifteen ng RNA per reaction were used to run the qPCR using pre-designed Taqman probes from Invitrogen/Applied Biosystems.

### 2-Deoxy-[^3^H]glucose uptake and cell surface GLUT4*myc*

L6GLUT4*myc* myoblasts grown in 24-well plates were serum-deprived for 2 h and then treated with or without insulin (100 nM, 20 min). 2-Deoxy-^3^H]glucose uptake and cell surface density of GLUT4*myc*[59] were measured as described previously.

### Immunoblotting

After treatments, cells were scraped into lysis buffer (20 mM Tris-HCl, 138 mMNaCl, 2.7 mM KCl, 1 mM MgCl, 2,5% glycerol and1% Nonidet-P40) supplemented with protease and phosphatase inhibitors (5 mM EDTA, 1 mM Na_3_VO_4_, 20 mM NaF, 1 mM dithiothreitol, protein inhibitor cocktail; Sigma-Aldrich) and protein content measured by the Bradford assay. For western blotting, proteins were boiled in Laemmli buffer, separated by SDS-PAGE and transferred onto PVDF membrane (Bio-Rad, Hercules, CA). Membranes were then blotted using primary antibodies (4°C overnight), washed and peroxidase-coupled secondary antibody (1:10,000) was applied for 1 h at room temperature. Membranes were developed using enhanced chemiluminescence (ECL, Perkin Elmer), and films analyzed using NIH ImageJ software.

### Intracellular reactive oxygen species (ROS) production

Cytoplasmic ROS level in L6GLUT4*myc* were assayed using the 5-(and-6)-chloromethyl-2',7'-dichlorodihydrofluorescein diacetate acetyl ester (CM-H2DCFDA, Invitrogen). Cells grown in 12-well plates were treated with 0.2 mM palmitate, palmitoleate or BSA control for 24 h, then incubated with 10 μM CM-H2DCFDA at 37°C for 30 min in PBS. H_2_O_2_ (100 μM) was used as positive control and it rendered a consistent 3-fold increase in ROS. Cells were washed and solubilized in Triton-X100 1% (v/v in distillated water) and fluorescence measured in a plate reader at 495 nm excitation and 520 nm emission. Results were normalized to the protein content of cell lysates.

### Generation of muscle conditioned medium

L6GLUT4*myc* cells grown to pre-confluence were treated with 0.2 mM palmitate, palmitoleate or BSA control in αMEM supplemented with 10% FBS, for 24 h. The fatty acids and BSA solutions were then removed, cell monolayers thoroughly rinsed with PBS and incubated with fresh αMEM supplemented with 2% FBS for another 24 h. The supernatants collected after this step represent the conditioned media (CM) and depending on whether muscle cells were pretreated with BSA, palmitate or palmitoleate, the CM are termed CM-BSA, CM-PA or CM-PO. CM were centrifuged at 10,000 RPM for 5 min to pellet debris, aliquoted and immediately frozen at -80°C. At the end of these incubations, cell viability in the corresponding cell monolayers was >85% (MTT test). Quantification of non-esterified fatty acids in CM was performed using the Non-Esterified Fatty Acid-HR kit (Wako) as per the manufacturer’s instructions.

### Characterization and inactivation of muscle conditioned medium

Cytokines in the CM were determined using the rat cytokine profiler array 2 (Ray Biotech) as per the manufacturer’s instructions. Signal detection was done using x-ray films and care was taken to expose the CM-BSA and CM-PA arrays equally.

CM were either heated at 95°C for 15 or treated with 100μg/mL proteinase K (Sigma Aldrich) for 2 h, then heat inactivated as above to avoid damage of macrophages by proteinase K.

### Immunofluorescence staining and cell shape score

All reagents were diluted in PBS supplemented with calcium and magnesium, and cells were washed with PBS between each staining step. Cells cultured on glass coverslips were fixed with 3% paraformaldehyde for 30 min and incubated sequentially with 0.1% Triton-X100 for 10 min, 5% BSA for 30 min, and rhodamine-phalloidin for 45 min. Nuclei were counterstained with 1 μg/ml DAPI (Sigma Aldrich) for 5 min. Coverslips were then mounted onto glass slides and stored at 4°C until analysis. Fluorescence images were captured on a spinning disk confocal microscope using a 40X air objective. For an analysis unbiased by cell shape, fields were selected by viewing nuclear staining (DAPI). Subsequent, rhodamine fluorescence was captured in x, y and z axes to detect actin within the whole cell volume. At least 300 cells were used for analysis per condition (>30 fields).

### Shape quantification

For each field, extended focus images (fusion of all z stack images) were created and image analysis was performed using ImageJ software (http://rsb.info.nih.gov/ij). For each field, the image was transformed in 8 bits and a median filter (2 pixel radius) was applied to approximate the distribution of staining intensity. Binary image masks were then created using an automatic threshold to create an image including all fluorescence data above background. Detection of surface area was done using of the “Analyze Particles” option with a threshold of 100 pixels in order to exclude all small dots of fluorescence outside of cells. Quantitative fluorescence data (area, circularity) were exported from ImageJ for further analysis and presentation. Area results given in pixel were then transformed in μm^2^ according to the scale. Additionally, the elongated *vs.* round phenotype was determined by manual counting of the cells and eye determination of the shape. This quantification was done by a sample identity-blinded experimenter to avoid subjective bias.

### Adhesion assay

RAW macrophages were pre-stained with calcein-AM (1 μg/ml) for 20 min and then centrifuged, washed and re-suspended in each test CM from the muscle cell cultures. After 30 min, macrophages were seeded onto a 24-well plate and allowed to adhere for 15 min. Medium was then removed, cells were washed and the remaining adherent cells were lysed in 1% Triton-X100. Fluorescence at excitation/emission of 495/515 nm was proportional to the number of adhered cells.

### Statistical analysis

Statistical analysis was performed using GraphPad Prism software (GraphPad Software, San Diego, CA). Two-groups comparison was performed using Student's paired t-test with Welsh correction for inequality of variances as necessary. Results of time courses were analyzed by two-way analysis of variance followed as appropriate by Bonferroni post-tests. One-way ANOVA was used to test differences between groups. Data are presented as means ± SD (if n=3) or SEM (if n≥4), and statistical significance was set at P < 0.05.

## Abbreviations

BSA: Bovine serum albumin; CM: Conditioned media; CM-BSA: Conditioned media from BSA-pretreated myoblasts; CM-PA: Conditioned media from palmitate-pretreated myoblasts; CM-PO: Conditioned media from palmitoleate-pretreated myoblasts; HFD: High fat diet; IκBα: Inhibitor of nuclear factor kappa-B; IL: Interleukin; INF: Interferon; NF-κB: Nuclear factor kappa-B; PA: Palmitate; PO: Palmitoleate; ROS: Reactive oxygen species; TNF: Tumor necrosis factor.

## Competing interests

The authors declare that they have no competing interests.

## Authors’ contributions

NJP participated in the design of the study, coordinated and carried out the majority of the experiments, performed the statistical analysis and participated in writing of the manuscript. KA carried out experiments and analysis concerning macrophage signalling, spreading and adhesion. PJB participated in the design of the study, helped with the experiments and writing of the manuscript. TTC carried out experiments involving gene silencing and surface GLUT4 detection. AK conceived the study and participated in its design, coordination, and writing of the manuscript. All authors read and approved the final manuscript.

## Supplementary Material

Additional file 1 **Figure S1.** Cytokine composition of the CM. CM-BSA and CM-PA were tested for their cytokine and chemokine content using commercially available rat profiler arrays from Ray Biotech. C) Map of the cytokines detected by the membrane. **Figure S2.** Palmitate-induced cytokine expression occurs through NFκB pathway. A) NFκB was silenced using specific siRNA to the p65 subunit (p65) or control non-related siRNA (NR) before treatment of cells for 24h with 0.2 mM palmitate or BSA. B) TLR2 and TLR4 were inhibited using a cell-permeant MYD88 inhibitory peptide (MYD-Inh) and results compared to a control scramble peptide. IL-6 expression was then measured by qPCR as previously. Inset: IL-6 expression measured in response to BSA and 10 ng/mL LPS for 24 h in presence or not of the MYD88 inhibitory peptide, expressed relative to the BSA control. All results were reported as fold change, relative to BSA ± SD from at least 3 independent experiments (n≥3). *P<0.05, ** P< 0.01, ns = not significant. **Figure S3.** Inactivation of the CM prevents TNFα expression in macrophages. A) CM from L6GLUT4myc cells was generated as described in methods and inactivated using boiling (95°C, 15 min) or treatment with proteinase K (100 μg/mL for 2 hours at 40°C followed by heat inactivation of the enzyme at 95°C for 15 min). CM was then tested for its ability to induce TNFα expression in RAW264.7 macrophages. Gene expression was measured by qPCR as described in Methods. Results were reported as a ratio over the CM-BSA control, mean ± SEM from 5 independent experiments (n=5). B) Results from the same experiments were expressed as a ratio PA over BSA. *P<0.05 versus BSA control. **Figure S4.** Inhibition of ERK prevents macrophage adhesion. RAW cells were pretreated with 20 μM PD98059 for 30 minutes. Measurement of RAW cells adhesion in response to the CM was then performed as described in methods. Results were reported as % of the CM-BSA control, mean ± SD from 4 independent experiments (n=4). *P<0.05, ns = not significant. (PPTX 684 kb) Clik here for file
