# Supplementary material for: Muscle cells challenged with saturated fatty acids mount an autonomous inflammatory response that activates macrophages
Source: Cell Commun Signal. 2012 Oct 19;10:30. doi: 10.1186/1478-811X-10-30 (PMC3507850; doi:10.1186/1478-811X-10-30)
Supplement: Additional file 1 — Figure S1. Cytokine composition of the CM. CM-BSA and CM-PA were tested for their cytokine and chemokine content using commercially available rat profiler arrays from Ray Biotech. C) Map of the cytokines detected by the membrane. Figure S2. Palmitate-induced cytokine expression occurs through NFκB pathway. A) NFκB was silenced using specific siRNA to the p65 subunit (p65) or control non-related siRNA (NR) before treatment of cells for 24h with 0.2 mM palmitate or BSA. B) TLR2 and TLR4 were inhibited using a cell-permeant MYD88 inhibitory peptide (MYD-Inh) and results compared to a control scramble peptide. IL-6 expression was then measured by qPCR as previously. Inset: IL-6 expression measured in response to BSA and 10 ng/mL LPS for 24 h in presence or not of the MYD88 inhibitory peptide, expressed relative to the BSA control. All results were reported as fold change, relative to BSA ± SD from at least 3 independent experiments (n≥3). *P<0.05, ** P< 0.01, ns = not significant. Figure S3. Inactivation of the CM prevents TNFα expression in macrophages. A) CM from L6GLUT4myc cells was generated as described in methods and inactivated using boiling (95°C, 15 min) or treatment with proteinase K (100 μg/mL for 2 hours at 40°C followed by heat inactivation of the enzyme at 95°C for 15 min). CM was then tested for its ability to induce TNFα expression in RAW264.7 macrophages. Gene expression was measured by qPCR as described in Methods. Results were reported as a ratio over the CM-BSA control, mean ± SEM from 5 independent experiments (n=5). B) Results from the same experiments were expressed as a ratio PA over BSA. *P<0.05 versus BSA control. Figure S4. Inhibition of ERK prevents macrophage adhesion. RAW cells were pretreated with 20 μM PD98059 for 30 minutes. Measurement of RAW cells adhesion in response to the CM was then performed as described in methods. Results were reported as % of the CM-BSA control, mean ± SD from 4 independent experiments (n=4). *P<0.05, ns = not [file 1478-811X-10-30-S1.pptx]

## Slide 1
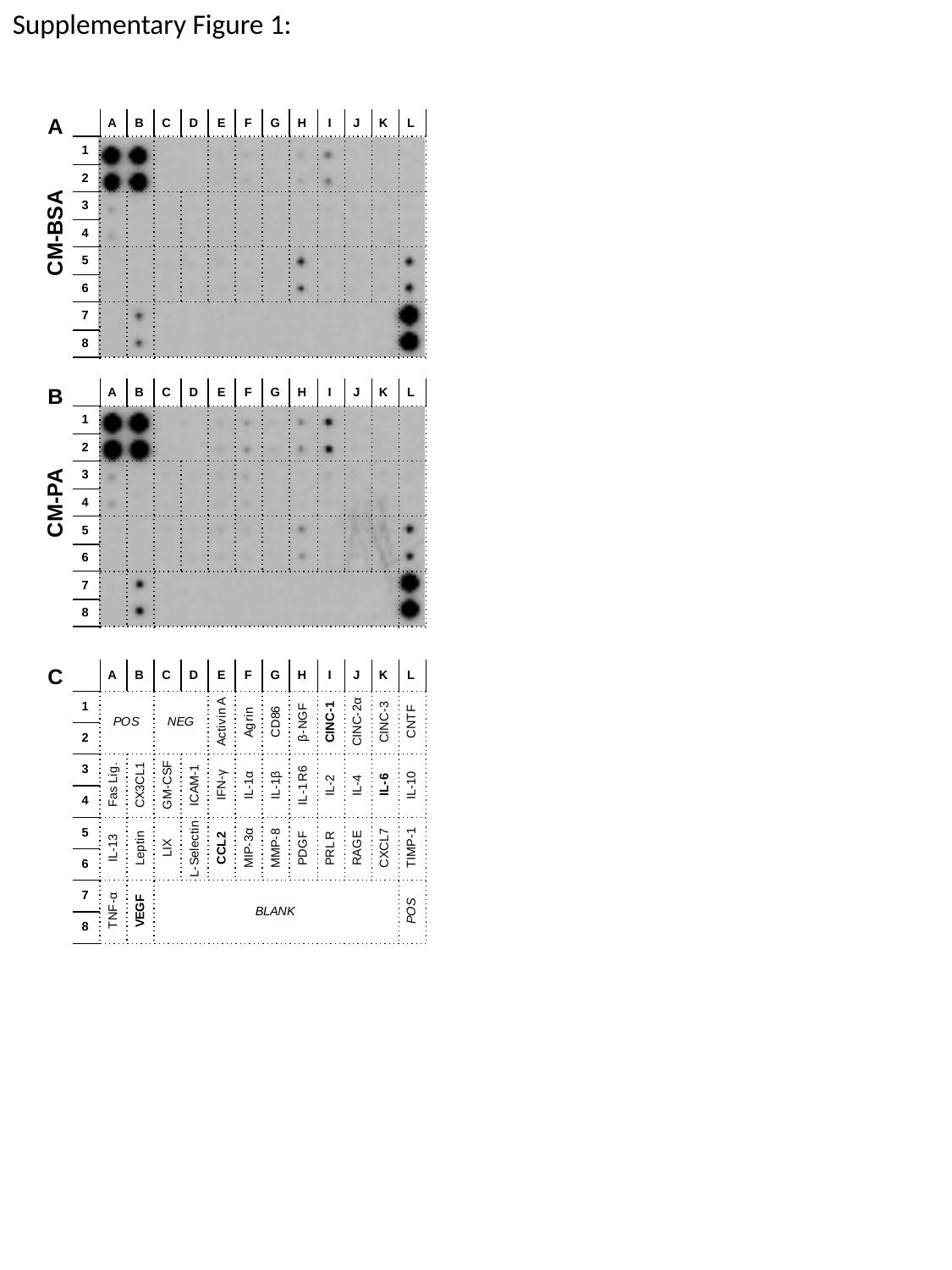

Supplementary Figure 1:

## Slide 2
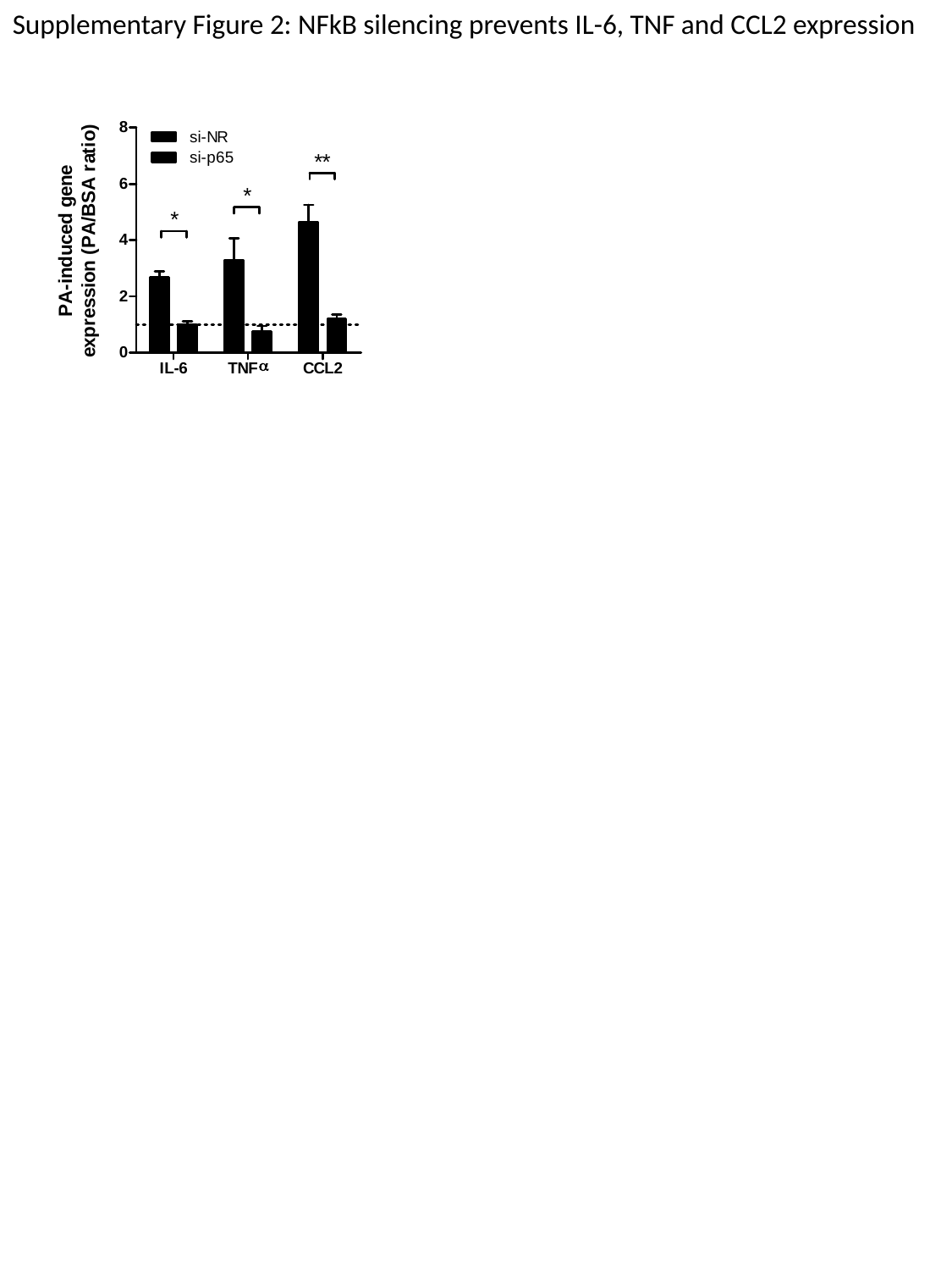

Supplementary Figure 2: NFkB silencing prevents IL-6, TNF and CCL2 expression

## Slide 3
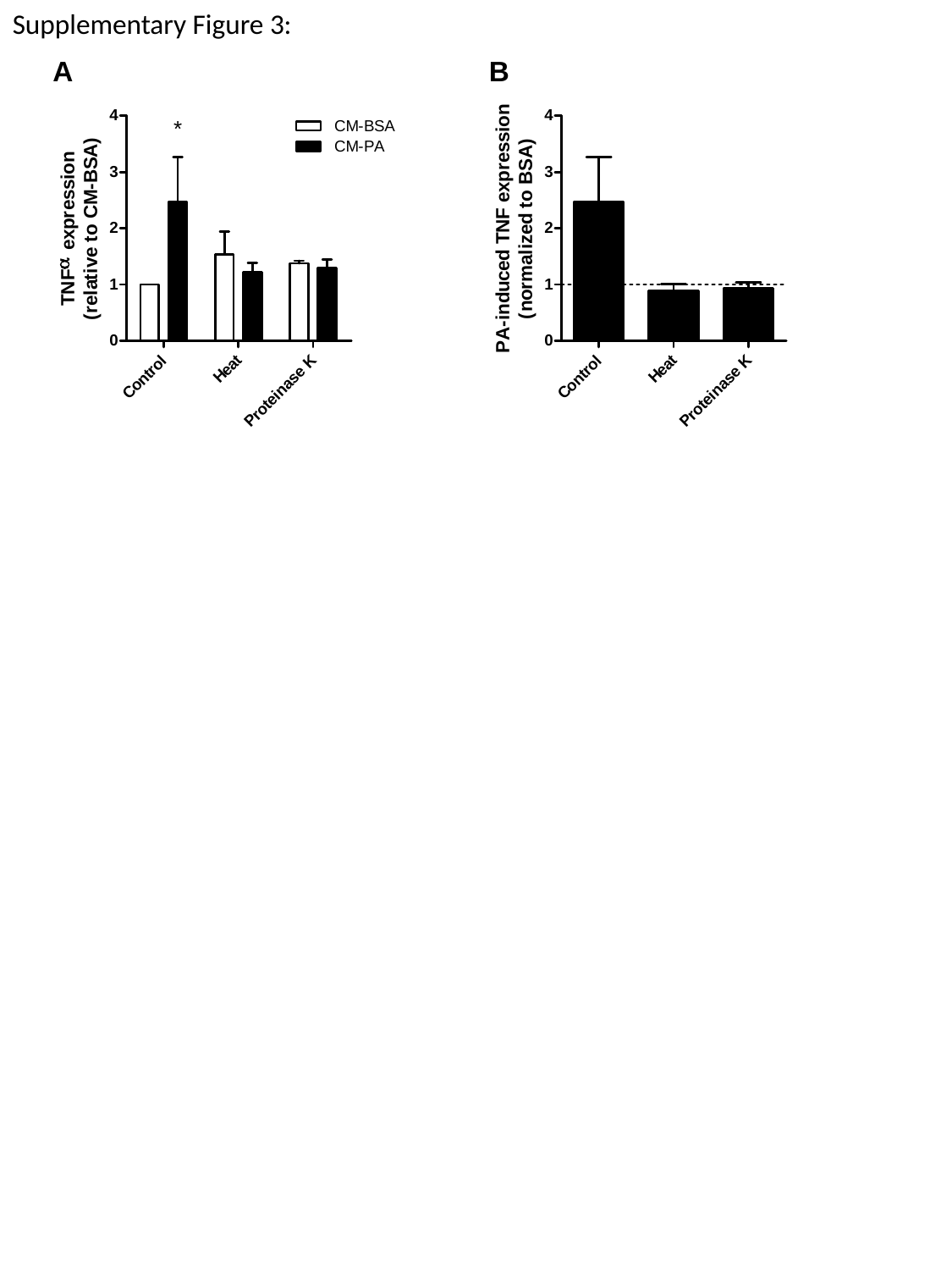

Supplementary Figure 3:
A
B

## Slide 4
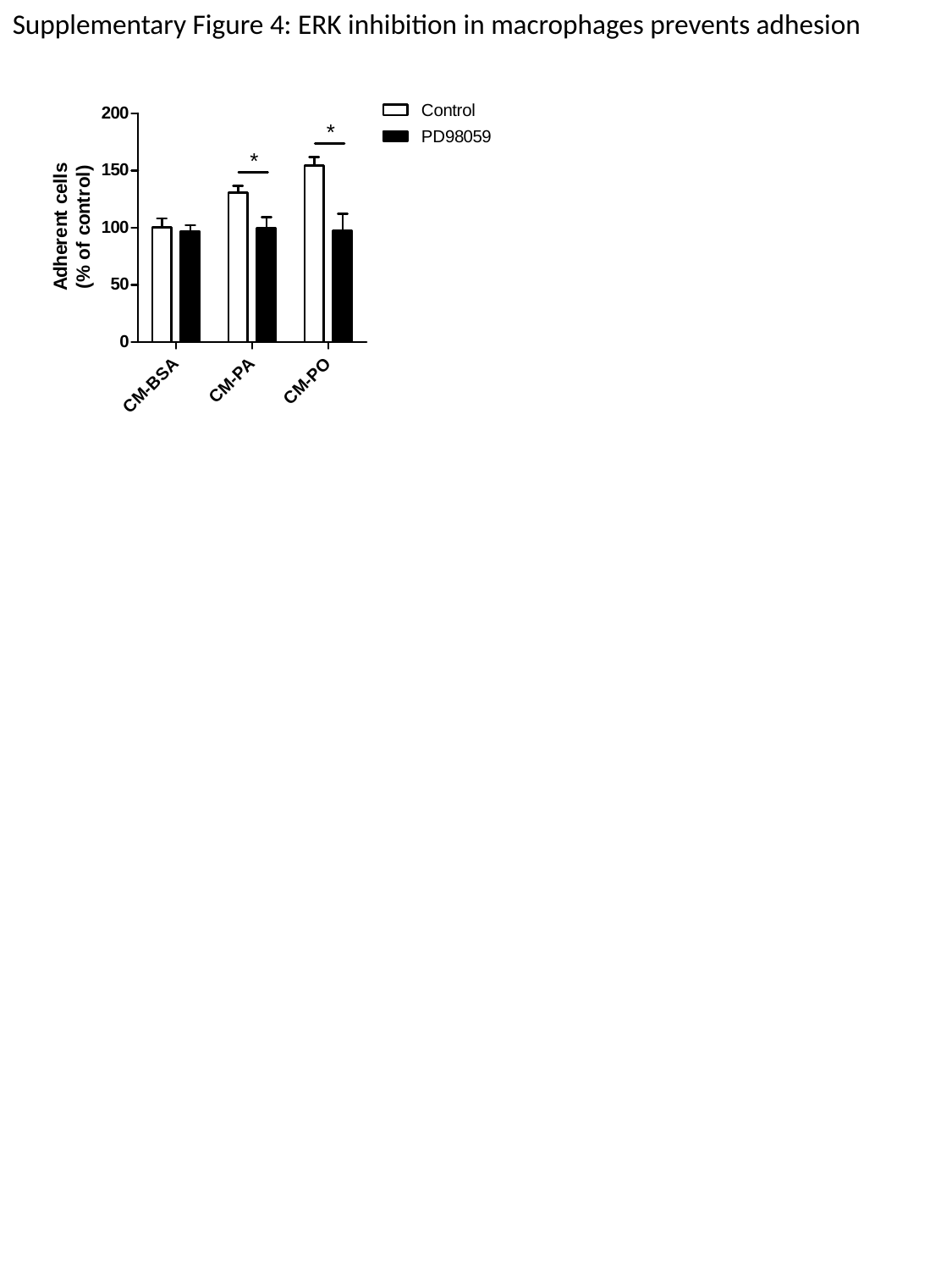

Supplementary Figure 4: ERK inhibition in macrophages prevents adhesion
